# Sodium Tanshinone IIA Sulfonate Enhances Effectiveness Rt-PA Treatment in Acute Ischemic Stroke Patients Associated with Ameliorating Blood-Brain Barrier Damage

**DOI:** 10.1007/s12975-017-0526-6

**Published:** 2017-02-27

**Authors:** Biying Ji, Fei Zhou, Lijuan Han, Jun Yang, Haijian Fan, Shanshan Li, Jingwei Li, Xin Zhang, Xiaoying Wang, Xiangyan Chen, Yun Xu

**Affiliations:** 1 Department of Neurology and Radiology, Drum Tower Hospital, Medical School and The State Key Laboratory of Pharmaceutical Biotechnology and Jiangsu Key Laboratory for Molecular Medicine of Nanjing University, Nanjing, People’s Republic of China; 2 GE Healthcare, Shanghai, People’s Republic of China; 3 Departments of Neurology, Massachusetts General Hospital, Harvard Medical School, Charlestown, MA USA; 4 Departments of Medicine and Therapeutics, Chinese University of Hong Kong, Shatin, Hong Kong, SAR China

**Keywords:** Blood-brain barrier, Sodium tanshinone IIA sulfonate, Thrombolysis, CT perfusion, Permeability surface

## Abstract

Treatment with sodium tanshinone IIA sulfonate (STS) may ameliorate blood-brain barrier (BBB) damage in acute ischemic stroke patients receiving recombinant tissue plasminogen activator (rt-PA) thrombolysis and improve stroke patients’ outcome. This randomized, single-center, placebo-controlled clinical trial investigated the potential effects and underlying mechanisms of STS. Forty-two acute ischemic stroke patients receiving intravenous rt-PA thrombolysis were randomized to intravenous administration either with STS (60 mg/day) (*n* = 21) or with equivalent volume of saline as a placebo (*n* = 21) after randomization for 10 days. Clinical outcomes, computer tomography perfusion (CTP) imaging with permeability-surface area product (PS) maps and serum levels of BBB damage biomarkers, were compared between the two groups. The percentage of patients with excellent functional outcome indicated by a 90-day mRS ≤1 was significantly higher in the STS group than in the placebo group (*p* = 0.028). For patients with CTP imaging (*n* = 30), PS in the ipsilateral lesion (*p* = 0.034) and relative PS (*p* = 0.013) were significantly lower in the STS group than that in placebo. STS-treated patients also had lower levels of matrix metalloproteinase (MMP)-9 (*p* = 0.036) and claudin-5 (*p* = 0.026), but higher levels of tissue inhibitor of metalloproteinase (TIMP)-1 (*p* = 0.040) than those in the placebo group. Post-stroke STS treatment could improve neurologic functional outcomes for acute ischemic stroke patients following rt-PA treatment by reducing BBB leakage and damage, which might be mechanistically associated with MMP-9 inhibition.

## Introduction

Blood-brain barrier (BBB) breakdown is a major contributing factor to ischemic brain injury or hemorrhagic transformation (HT) and often leads to poor outcomes in acute ischemic stroke patients receiving recombinant tissue plasminogen activator (rt-PA) treatment [1, 2]. Tight-junction proteins such as claudin, junctional adhesion molecule, and occludin play essential roles in maintaining BBB integrity. Measurements of permeability-surface area product (PS), an indicator of BBB permeability, by computer tomography perfusion (CTP) imaging have been successfully applied for early identification of BBB damage and HT development in acute stroke patients [3, 4].

Sodium tanshinone IIA sulfonate (STS) is a water-soluble derivative of tanshinone IIA, a main bioactive component isolated from the roots of the Chinese herb *Salviae miltiorrhiza* Bunge (Danshen) [5]. STS has been widely used for treatments of cardiovascular and cerebrovascular diseases in China [6, 7]. In mice with cerebral ischemia, STS could protect BBB and had a patent in China [8, 9]. Here, we hypothesized that STS could work as a BBB protective agent that help acute ischemic stroke patients who received thrombolysis treatment recover better. In this study, we used CTP-derived PS to reveal whether treatment with STS could reduce BBB leakage in acute ischemic stroke patients receiving rt-PA thrombolysis and sought to investigate the underlying mechanisms.

## Materials and Methods

### Participants

**Fig. 1:**
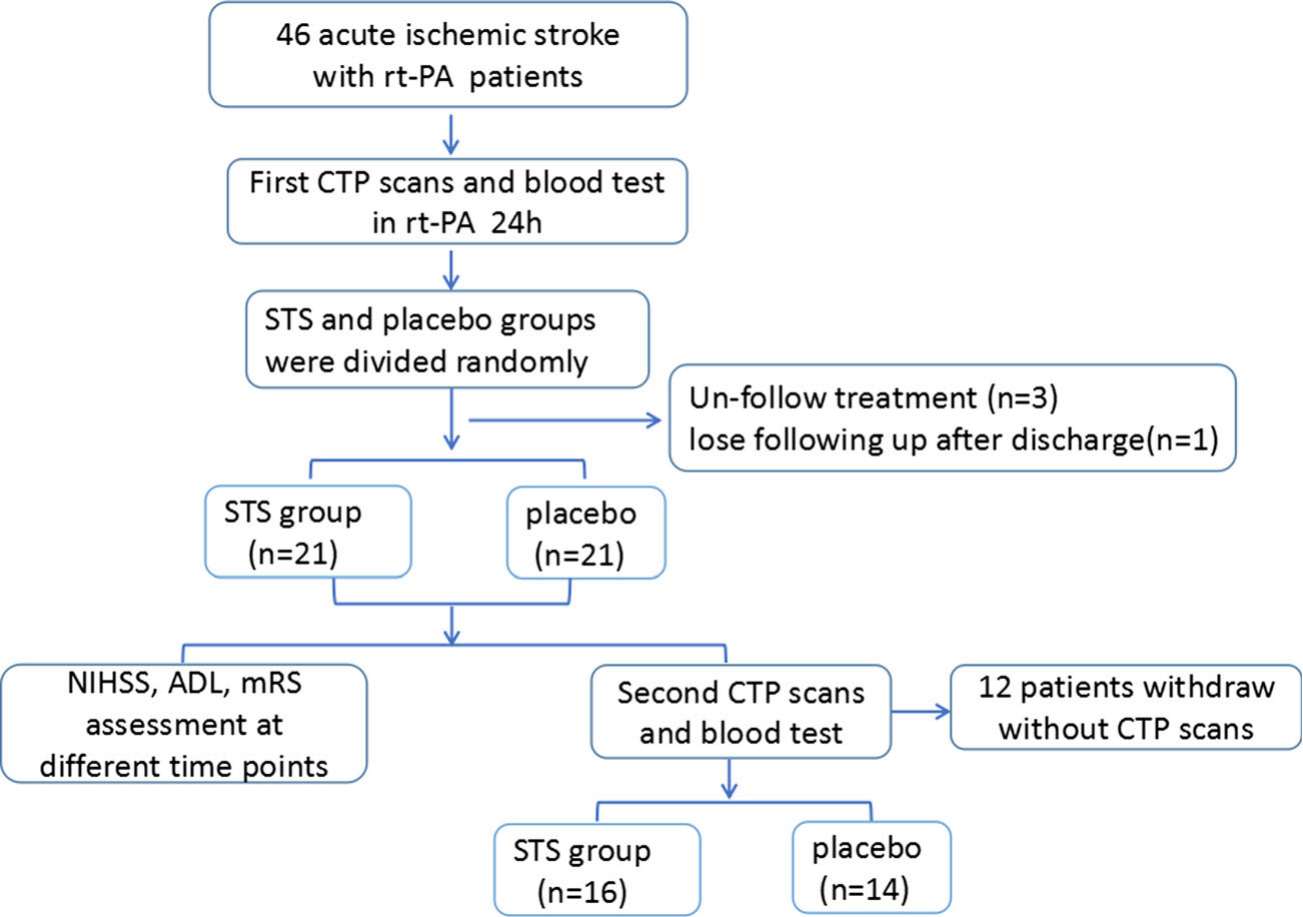
Flow chart showing patient cohort selection in this study

This single-centered, randomized, double-blinded prospective study was approved by the ethics committee of Drum Tower Hospital, Medical School of Nanjing University. An entry was made in the Chinese clinical trial registry (ChiCTR-ONRC-14004659). The inclusion criteria were as follows: (1) acute ischemic stroke patients receiving rt-PA treatment, (2) at age between 18 to 80 years old, and (3) willing to participate in all follow-up neurologic and imaging examinations. We excluded patients with presence of platelet abnormalities (PLT < 100 or >300 × 10^9^/L), severe bleeding disorders, contraindications to iodinated contrast agent, a history of severe renal failure, or known or suspected infection. Informed consents were obtained from all participants involved in this study. Randomization sequences were computer generated. The flow chart of patient cohort selection is shown in Fig. 1.

### Treatments

After rt-PA thrombolysis, STS (60 mg per day) was intravenously administrated daily to patients in the STS group for 10 days, while patients in the placebo group received equivalent administrations of saline. All participants received aspirin after thrombolysis 24 h for acute ischemic stroke.

### Neurologic Assessment

Neurologic function outcomes were assessed using the National Institutes of Health Stroke Scale (NIHSS) score at 0, 24 h, and 10 days; activities of daily living (ADLs) score at 0 h and 10 days; and the modified Rankin Scale (mRS) at 90 days.

### CTP Scan Protocol and Image Analysis

CT perfusion and non-contrast CT scans were performed on a 64-slice CT scanner (Discovery CT750 HD, GE Healthcare, Milwaukee, WI, USA). CTP started with intravenous injection of 50 mL of iodinated contrast agent (350 mg/mL, Omnipaque, GE Healthcare, Shanghai, China) followed by a saline flush of 45 mL at 5 mL/s. CTP scans began after a delay of 5 s from contrast injection, and the following technical settings were applied: 80 kVp, 150 mAs, temporal sampling rate of 2 s for 60 s, and total axial coverage of 40 mm at 5-mm slice thickness.

All CTP images were analyzed by experienced radiologists who were blinded to this research. For the PS maps and data, manually drawn region of interests (ROIs) in the ipsilateral hemisphere were compared to those in the contralateral hemisphere. PS values were calculated using CTP software (CT Kinetics, GE Healthcare, China). The corresponding ROIs for the contralateral side were generated automatically by mirroring the ipsilateral ROIs, and the relative PS (rPS) was defined as ipsilateral/contralateral ROIs.

### Measurement of BBB Damage Biomarkers

Venous blood were collected at 0, 24 h, and 10 days, and the separated serum was stored in aliquots at −80 °C until biochemical analysis.

Serum levels of matrix metalloproteinase (MMP)-2 (R&D, Minneapolis, IL, USA), MMP-9 (R&D, Minneapolis, IL, USA), tissue inhibitor of metalloproteinase (TIMP)-1 (R&D, Minneapolis, IL, USA), claudin-5 (CusaBio, Wu han, China), and zonula occluden (ZO)-1 (CusaBio, Wu han, China) were measured using commercially available ELISA kits according to the manufacturer’s instructions.

### Study Outcomes

The outcomes were the integrity of blood-brain barrier by measurement of PS, MMP-9, MMP-2, TIMP-1, claudin-5, and ZO-1 and neurologic improvement by the score on NIHSS and the mRS at 90 days. Safety outcome measures the incidence of adverse event.

### Statistical Analysis

All results were analyzed using SPSS (SPSS version 22.0, Chicago, Illinois, USA). The data were shown as mean ± standard deviation (SD) or medians with interquartile ranges for continuous variables and proportions for categorical variables. Continuous variables were compared using *t* tests, and categorical variables were analyzed using the Pearson *χ* ^2^ test or Fisher’s exact test. *P* value <0.05 was considered statistically significant.

## Results

### Patient Characteristics

**Fig. 2:**
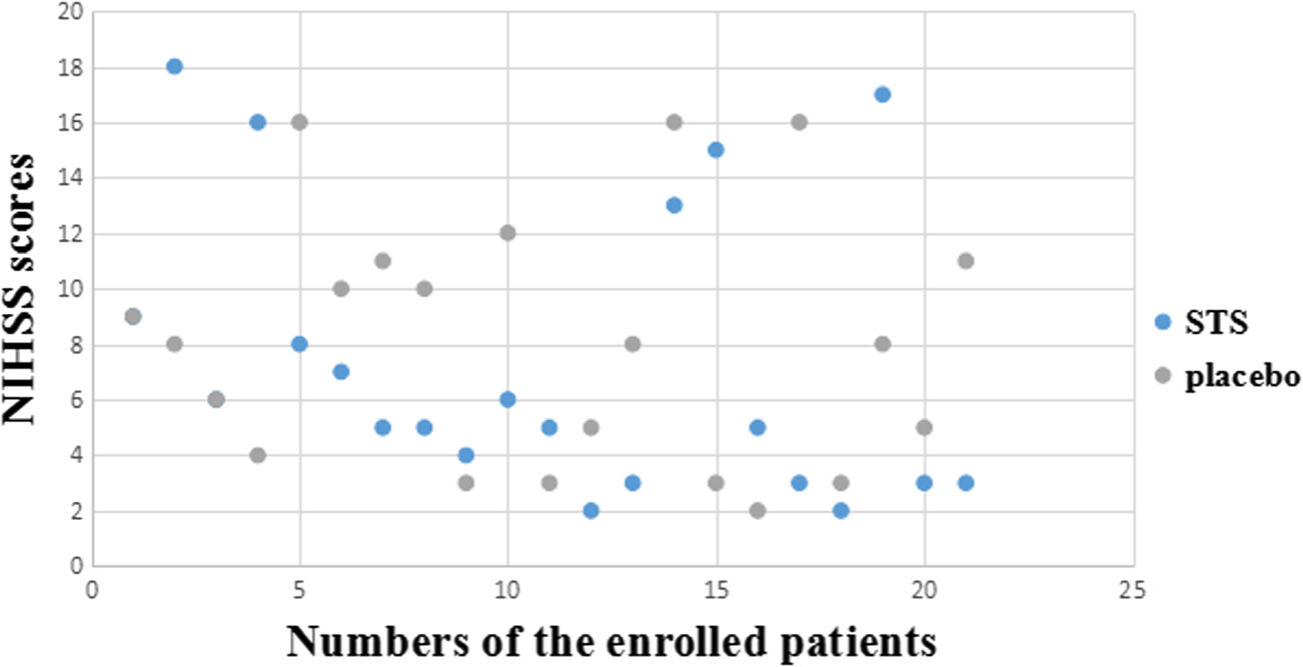
The distribution of baseline NIHSS between the two groups

From February 2014 to February 2016, 46 patients receiving rt-PA were included: 23 patients were treated with STS and 23 with placebo as control. Three patients had uncompleted treatment due to transferring to another department, and one patient was lost follow-up, thus leaving 21 patients in each group for clinical and prognostic analysis. General clinical characteristics (e.g., age, sex, risk factors, and NIHSS scores) and the distribution of baseline NIHSS at admission were similar (Table 1 and Fig. 2). In the 42 patients, 12 were unavailable to attend the second CT examinations, leaving 16 and 14 patients for imaging analysis in the STS and placebo groups, respectively.

**Table 1:** Patients characteristics

	STS (*N* = 21)	Placebo (*N* = 21)	F/χ^2^	*P* value
Average age (years)	63.81 ± 9.872	63.62 ± 11.561	1.321	0.954
Male (%)	14 (66.7%)	14 (66.7%)	0.000	1.000
Hypertension	12 (57.1%)	17 (81.0%)	2.785	0.181
Diabetes	3 (14.3%)	5 (23.8%)	0.618	0.694
Hyperlipidemia	7(33.3%)	6 (28.6%)	0.111	1.000
Hyperhomocysteinemia	5 (23.8%)	4 (19.0%)	0.141	1.000
Atrial fibrillation	4 (19.0%)	2 (9.5%)	0.778	0.663
Coronary heart disease	3 (14.3%)	1 (4.8%)	1.105	0.606
Previous cerebral infarction	2 (9.5%)	3 (14.3%)	0.227	1.000
Smoking	5 (23.8%)	3 (14.3%)	0.618	0.238
Alcohol use	6 (28.6%)	2 (9.5%)	2.471	0.238
At admission				
SBP (mmHg)	153.48 ± 17.665	154.81 ± 21.558	1.896	0.828
DBP (mmHg)	86.62 ± 14.333	85.29 ± 13.050	0.527	0.754
Glu (mmol/L)	7.93 ± 2.595	8.66 ± 4.070	6.424	0.492
Time to rt-PA (min)	214.62 ± 43.246	198.71 ± 42.717	0.021	0.238
NIHSS	8.05 ± 4.522	7.38 ± 5.220	0.427	0.661
ADL	54.29 ± 25.801	53.33 ± 27.034	0.054	0.908

*SBP* systolic blood pressure, *DBP* diastolic blood pressure

### Clinical Outcomes

**Fig. 3:**
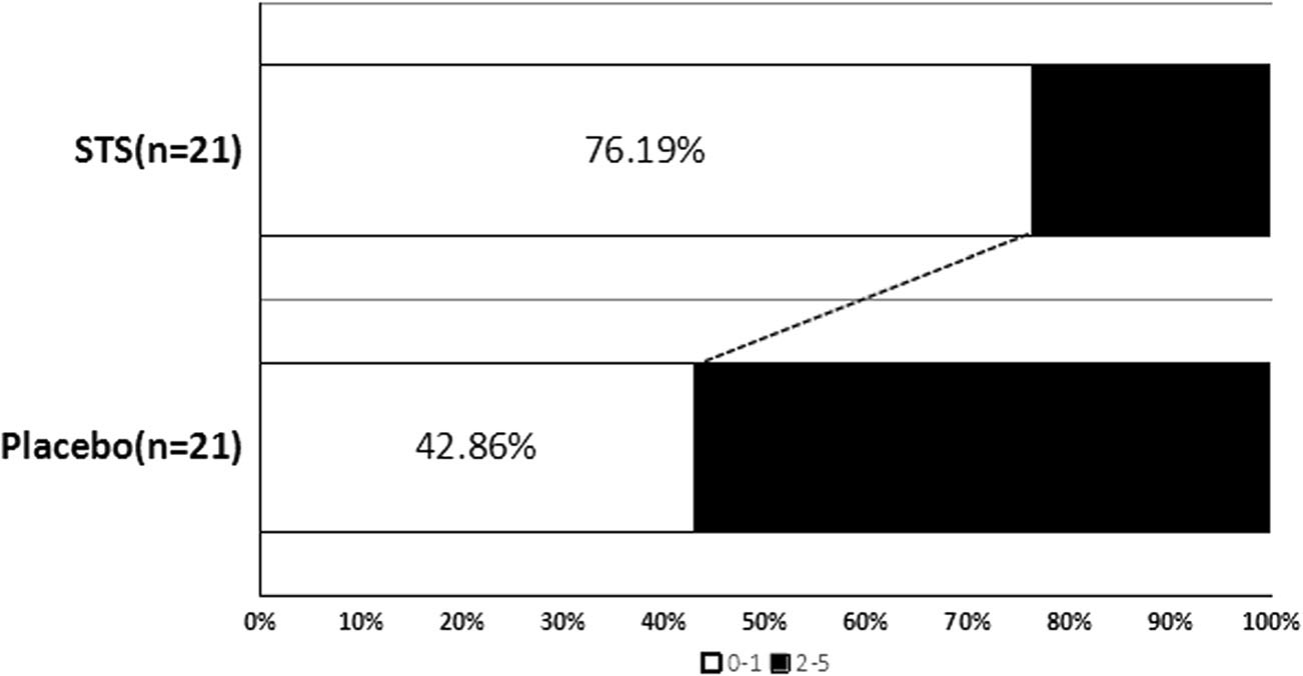
Distribution of mRS in the STS and placebo groups at 3 months after treatment. STS had more patients with 90-day mRS score ≤1 in the whole 42 patients

A battery of neurologic function assessments was used to investigate whether patients with acute ischemic stroke could benefit from STS after rt-PA thrombolysis. Results showed that in whole 42 patients (21 STS and 21 placebo), there were more patients with a 90-day mRS score ≤1 (an excellent functional outcome) in the STS group than that in the placebo group. Though there is no statistical significance, STS did decrease the incidence of HT during hospitalization. No significant difference was found in the means of NIHSS and ADL score at 10 days after rt-PA treatment between the two groups (Table 2 and Fig. 3).

**Table 2:** Patient clinical outcomes

All enrolled patients	STS (*N* = 21)	Placebo (*N* = 21)	F/χ^2^	*P* value
10 day-NIHSS	2.81 ± 2.64	4.10 ± 3.81	1.853	0.211
10 day-ADL	82.62 ± 20.89	72.38 ± 25.18	1.016	0.159
90 day-mRS ≤ 1	16 (76.19%)	9 (42.86%)	6.462	0.028*
HT during hospitalization	2 (9.52%)	5 (23.81%)	1.543	0.410

*p<0.05

### Neuroimaging of BBB Permeability between STS and Placebo Groups

**Fig. 4:**
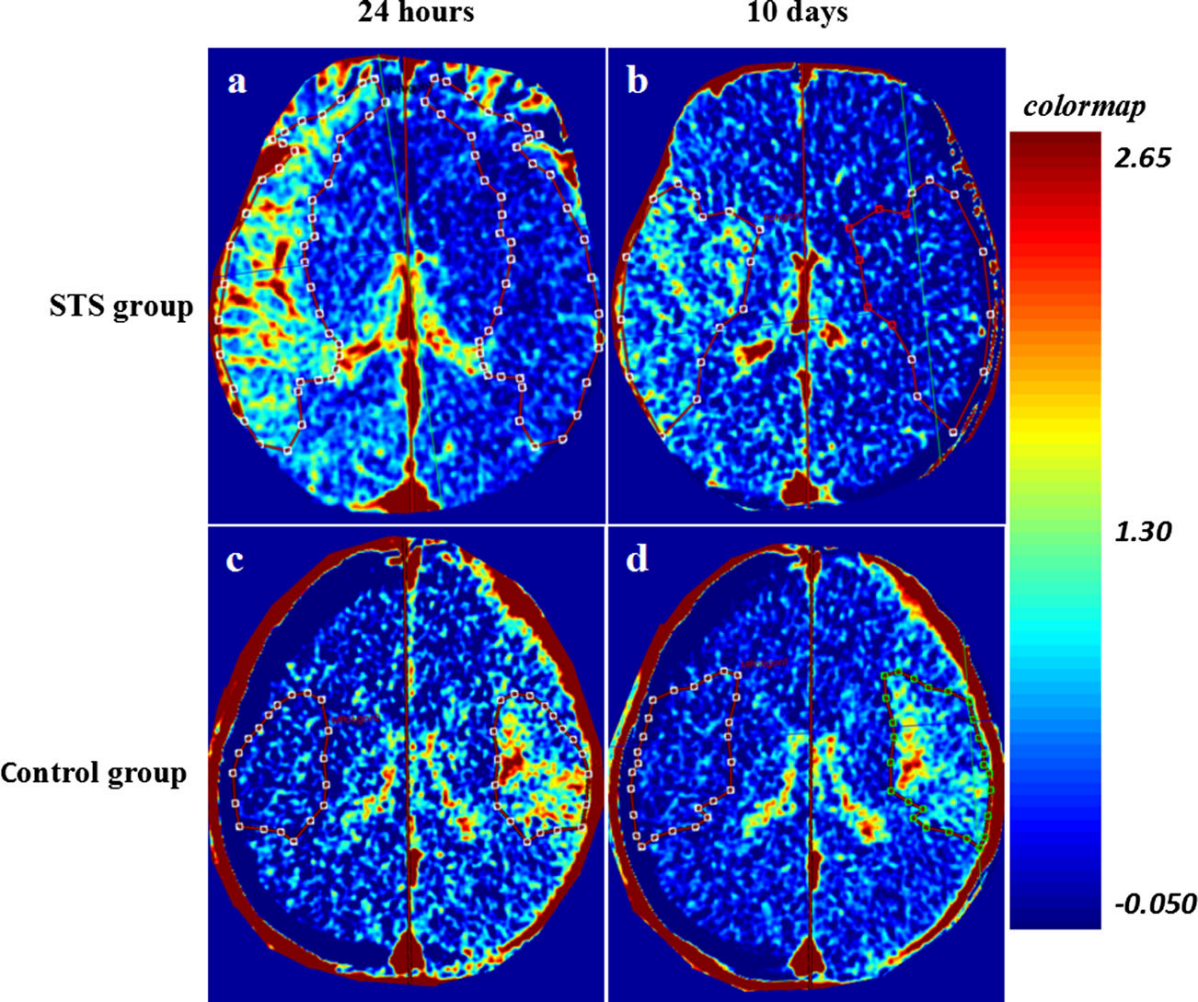
BBB-PS maps from the STS and placebo groups. Quantitative PS maps of both groups at baseline (**a**, **c**) and 10 days after STS or placebo treatment (**b**, **d**). At day 10, the patient with STS treatment showed a significant decline in BBB-PS when compared to the placebo

CTP was performed to reveal the effects of STS treatment on BBB integrity. Twenty-four hours after rt-PA treatment, a baseline CTP was performed for each patient and there was no significant difference in BBB permeability (ipsilateral or contralateral and rPS) between the two groups. The follow-up CTP was performed at 10 days after the treatments. Moreover, the STS group showed significantly lower levels of ipsilateral PS and rPS than did the placebo group at 10 days (Table 3 and Fig. 4).

**Table 3:** BBB permeability measured by CTP-derived PS

Parameters	STS (*N* = 16)	Placebo (*N* = 14)	*P* value
Baseline			
Ipsilateral PS value (ml/100 g/min)	0.373 ± 0.062	0.395 ± 0.073	0.371
Contralateral PS value (ml/100 g/min)	0.175 ± 0.025	0.179 ± 0.022	0.703
rPS	2.128 ± 0.219	2.225 ± 0.380	0.394
PS region area (cm^2^)	2.586 ± 2.461	2.893 ± 3.131	0.766
10 days after STS or placebo			
Ipsilateral PS value (ml/100 g/min)	0.266 ± 0.083	0.332 ± 0.079	0.034*
Contralateral PS value (ml/100 g/min)	0.170 ± 0.170	0.172 ± 0.017	0.761
rPS	1.548 ± 0.393	1.910 ± 0.345	0.013*
PS region area (cm^2^)	1.773 ± 1.563	2.287 ± 2.214	0.464
Infarct volumes (cm^3^)	2.020 ± 1.762	2.415 ± 3.083	0.676

### Serum BBB Damage Biomarkers

**Fig. 5:**
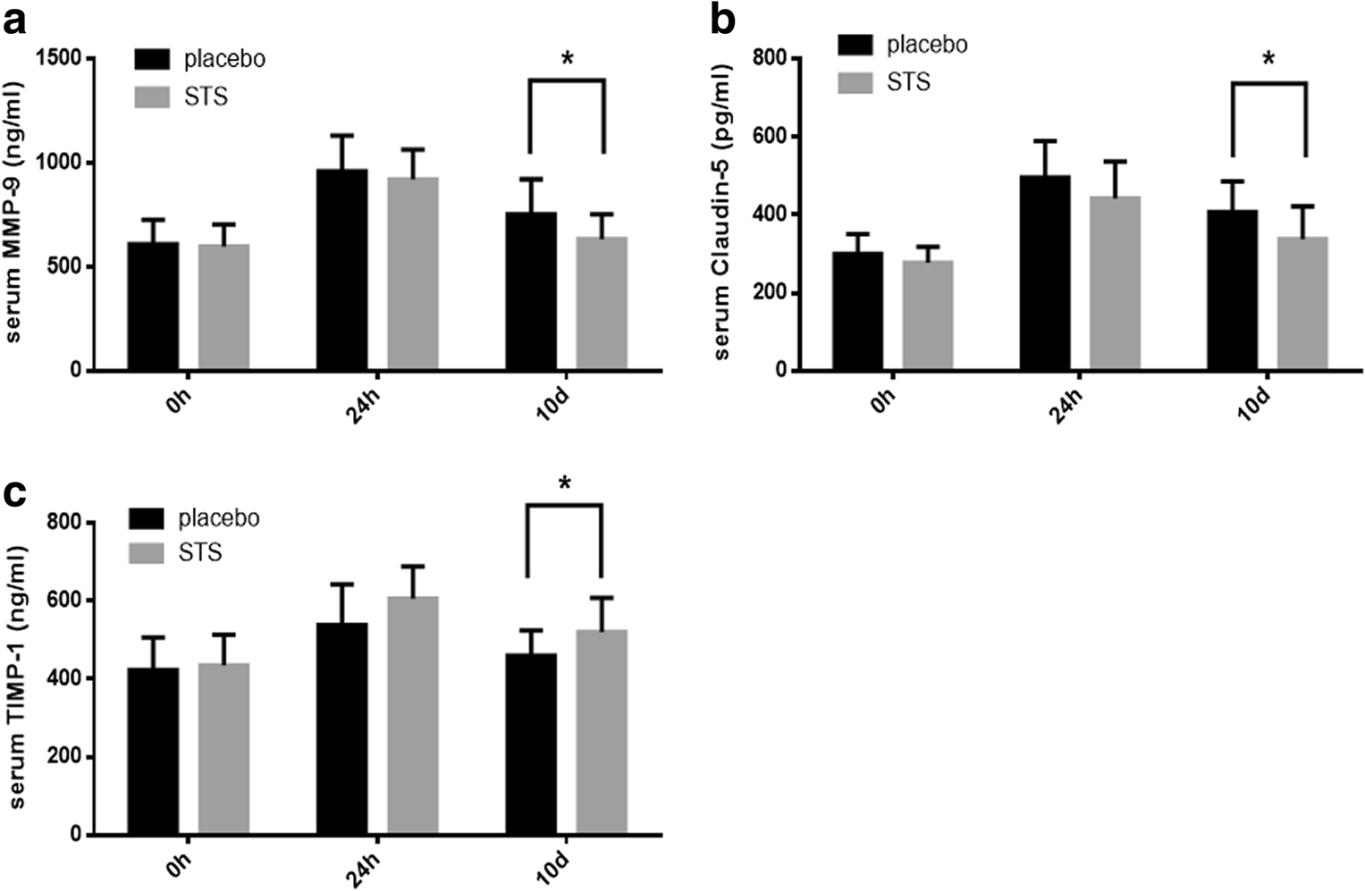
Serum levels of BBB damage biomarkers. Serum MMP-9 (**a**), claudin-5 (**b**), and TIMP-1 (**c**) protein levels were measured at different time points using ELISA in the STS (*n* = 16) and placebo groups (*n* = 14). STS showed a lower MMP-9 and claudin-5 and higher TIMP-1 expression after 10 days treatment

Serum levels of MMP-9, MMP-2, TIMP-1 and tight-junction proteins, including claudin-5 and ZO-1, in 30 patients (16 STS and 14 placebo) were measured to demonstrate BBB damage. The STS group had lower MMP-9 (633.352 vs 750.739 ng/ml, *p* = 0.036, Fig. 5a) and claudin-5 (337.822 vs 407.763 pg/ml, *p* = 0.026, Fig. 5b) levels but higher TIMP-1 expression (520.652 vs 459.567 ng/ml, *p* = 0.040, Fig. 5c) than did the placebo group at 10 days after thrombolytic therapy. However, at acute phase (0 and 24 h), there were no significant differences in all biomarkers between the two groups.

## Discussion

Current rt-PA thrombolytic therapy could augment BBB disruption in the acute stroke patients, which increase ischemic brain injury or HT [10]. Therefore, therapeutic strategies designed to alleviate BBB damage are needed to improve clinical outcomes of rt-PA thrombolysis [11]. Prognosis of rt-PA-treated patients with mRS ≤1 (an excellent functional outcome) was reported differently, from 42.7% to 54.6% at 3 months [12–14]. As Tsivgoulis reported, mRS ≤1 scores was 42.7%, which is similar to our control group (mRS ≤ 1 scores was 42.86%). Effect of rt-PA therapy is affected by many factors, such as treatment time since stroke onset, age, stroke severity, BBB integrity, and so on. Our study found that there were more patients with a 90-day mRS score ≤1 in the STS group compared with the placebo group.

To investigate whether the neuroprotection of STS is associated with decreasing BBB disruption, PS was detected by dynamic contrast-enhanced CT [10, 15, 16]. Recently, CTP-derived PS quantification has been used to predict BBB permeability and HT for acute stroke patients because of its good reproducibility in hemodynamic measurement, wide accessibility, and relatively low cost [2, 4, 10]. The neuroimaging results from this study showed that STS treatment could reduce BBB-PS to ameliorate BBB damage and benefit clinical outcomes post-rt-PA thrombolysis in acute stroke patients. Pathologically, release of tight junction adhesion molecules to blood circulation is associated with compromised BBB integrity in ischemic stroke [3]. Tanshinone IIA (precursor of STS) treatment has been previously shown to diminish BBB breakdown in experimental model of ischemic stroke [8, 9] and autoimmune encephalomyelitis [17]. Similarly, in this study, we found that STS treatment decreased the levels of BBB damage biomarkers, MMP-9 and claudin-5, in acute ischemic stroke patients after intravenous thrombolysis. From our observations, STS seemed to reduce the effects of BBB disruption by inhibiting MMP-9 activity and increasing expression of TIMP-1.

Next, we evaluated the infarct volumes and found no difference between the STS group (2.020 ± 1.762 cm^3^) and the placebo group (2.415 ± 3.083 cm^3^) after 10 days of treatment (*p* = 0.676). It suggested that STS did not reduce BBB damage through decreasing infarct volumes.

Furthermore, to study whether STS decreases cerebral hemorrhagic transformation, STS did reduce the trend of cerebral hemorrhagic transformation, but it was not significant, which may be correlated to the small sample research. STS could inhibit peripheral inflammatory cells into brain after stroke by suppressing BBB injury, which protects brain from immuno-inflammation and improves recover of stroke patients.

How STS protects the BBB from injury after stroke remains unclear. STS might improve BBB damage by suppressing astrocyte-mediated inflammation or decreasing brain microvascular endothelial cell apoptosis after stroke.

Together, the neuroimaging and pathological evidence of our study could delineate the mechanistic pathway of STS treatment in improving BBB dysfunction post-rt-PA thrombolysis.

However, we had several limitations in the current work. First, to better elucidate the relation between BBB damage biomarkers and PS, MMPs and tight junction proteins obtained from cerebrospinal fluid (CSF) examination by lumbar puncture and a correlation study are needed in the future. Second, lack of BBB-PS measurements from CTP imaging at 90 days could not fully evaluate the effects of STS treatment on BBB repair. In addition, our study had a small sample size although the randomization has been applied. These questions will be addressed in our future work.

In conclusion, both neuroimaging and serum biomarkers of BBB damage in this study demonstrated that acute ischemic stroke patients might benefit from STS treatment by ameliorating/diminishing BBB disruption following rt-PA thrombolysis.
